# ERK3 and DGKζ interact to modulate cell motility in lung cancer cells

**DOI:** 10.3389/fcell.2023.1192221

**Published:** 2023-05-23

**Authors:** Amanda K. Myers, Marion Morel, Stephen H. Gee, Katherine A. Hoffmann, Weiwen Long

**Affiliations:** ^1^ Department of Biochemistry and Molecular Biology, Wright State University, Dayton, OH, United States; ^2^ Department of Cellular and Molecular Medicine, University of Ottawa, Ottawa, ON, Canada; ^3^ Centre for Neuromuscular Disease, University of Ottawa, Ottawa, ON, Canada

**Keywords:** extracellular signal-regulated kinase 3 (ERK3), diacylglycerol kinase ζ (DGKζ), lung cancer, cell motility, cell membrane protrusion

## Abstract

Extracellular signal-regulated kinase 3 (ERK3) promotes cell migration and tumor metastasis in multiple cancer types, including lung cancer. The extracellular-regulated kinase 3 protein has a unique structure. In addition to the N-terminal kinase domain, ERK3 includes a central conserved in extracellular-regulated kinase 3 and ERK4 (C34) domain and an extended C-terminus. However, relatively little is known regarding the role(s) of the C34 domain. A yeast two-hybrid assay using extracellular-regulated kinase 3 as bait identified diacylglycerol kinase ζ (DGKζ) as a binding partner. DGKζ was shown to promote migration and invasion in some cancer cell types, but its role in lung cancer cells is yet to be described. The interaction of extracellular-regulated kinase 3 and DGKζ was confirmed by co-immunoprecipitation and *in vitro* binding assays, consistent with their co-localization at the periphery of lung cancer cells. The C34 domain of ERK3 was sufficient for binding to DGKζ, while extracellular-regulated kinase 3 bound to the N-terminal and C1 domains of DGKζ. Surprisingly, in contrast to extracellular-regulated kinase 3, DGKζ suppresses lung cancer cell migration, suggesting DGKζ might inhibit ERK3-mediated cell motility. Indeed, co-overexpression of exogenous DGKζ and extracellular-regulated kinase 3 completely blocked the ability of ERK3 to promote cell migration, but DGKζ did not affect the migration of cells with stable ERK3 knockdown. Furthermore, DGKζ had little effect on cell migration induced by overexpression of an ERK3 mutant missing the C34 domain, suggesting DGKζ requires this domain to prevent ERK3-mediated increase in cell migration. In summary, this study has identified DGKζ as a new binding partner and negative regulator of extracellular-regulated kinase 3 in controlling lung cancer cell migration.

## 1 Introduction

Diacylglycerol kinases (DGKs) are a family of enzymes that phosphorylate diacylglycerol (DAG) to yield phosphatidic acid (PA). The importance of these proteins is two-fold: not only do they terminate DAG signaling, but they also generate PA, which itself activates a different series of pathways. Mammals have ten different DGK isoforms, each the product of a separate gene, with DGKα and DGKζ being the most studied (Rincón et al., 2012). DGKs are classified into five subgroups according to their shared protein domains, with DGKζ and DGKι comprising the Type IV subgroup (Topham and Epand, 2009; Rincón et al., 2012). These DGKs contain two adjacent C1 domains (C1A and C1B), a myristoylated alanine-rich C-kinase substrate (MARCKS) domain, a catalytic domain, an ankyrin repeat domain, and a C-terminal PDZ-binding motif. The C1A and C1B regions are cysteine-rich domains like those found in protein kinase C (Topham and Epand, 2009). While the C1 domains in some DGK isoforms bind DAG, those in Type IV DGKs appear to have evolved to bind small GTPases and other proteins. For example, the C1A domain in DGKζ binds Rac1 (Yakubchyk et al., 2005).

The role of DGKζ in cell motility has been studied using mouse embryonic fibroblasts (MEFs) derived from wild-type (WT) and DGKζ-null mice (Zhong et al., 2003). In wound healing and Transwell^®^ migration assays, DGKζ-null MEFs migrated more slowly than WT MEFs when stimulated with platelet-derived growth factor. Abramovici et al. (2009) DGKζ-null cells were found to have less active PAK1 and less active Rac1, both of which are involved in cell motility, as compared with WT fibroblasts. Adding PA or a WT DGKζ—but not the kinase-dead protein—to the knockout cells rescued Rac1-GTP levels, suggesting DGKζ kinase activity is responsible for this effect. Interestingly, DGKζ was found to also activate RhoA in the same cell line in a kinase-independent manner by promoting PKCα-mediated phosphorylation of RhoGDI (Ard et al., 2012; 2021). The available data suggest DGKζ enhances cell motility. For example, the highly metastatic colon cancer cell line SW620 had increased DGKζ mRNA and protein levels, as well as increased invasion compared to the isogenic, less metastatic SW480 cell line (Cai et al., 2014). In addition, DGKζ silencing in SW620 cells reduced active Rac1 and cell invasion. DGKζ silencing also inhibited the invasion of both PC-3 (prostate) and MDA-MB-231 (breast) cancer cell lines.

Extracellular signal-regulated kinase 3 (ERK3), also known as MAPK6, is a protein kinase classified as an atypical mitogen-activated protein kinase (MAPK). It consists of three regions: an N-terminal kinase domain, a central conserved in ERK3 and ERK4 (C34) domain, and a unique C-terminal domain (Boulton et al., 1991; Coulombe and Meloche, 2007). Unlike the classical MAPKs, such as ERK1/ERK2, that contain a dual phospho-acceptor site within a Thr–Glu–Tyr motif in their kinase domain activation loop, the activation loop of ERK3 contains a single phospho-acceptor site (Ser189) within a Ser–Glu–Gly signature sequence (Cheng et al., 1996; Roskoski, 2012). In contrast to the classical MAPKs, ERK3 phosphorylation at the activation loop is not altered by typical mitogenic stimuli, such as serum or phorbol esters, though recent work suggests EGF stimulation or activating KRas mutation may increase ERK3 phosphorylation (Déléris et al., 2008; Bogucka et al., 2021; Bogucka-Janczi et al., 2023). The C34 domain and the unique C-terminal domain are not present in other MAPKs, and at present, their function is not well-understood (Coulombe and Meloche, 2007).

While the physiological functions of ERK3 still remain largely unknown, a role for ERK3 in promoting cell migration has been documented in a variety of cancer cell lines, including lung, breast, and head and neck cancer cell lines (Long et al., 2012; Al-Mahdi et al., 2015; Elkhadragy et al., 2017). SiRNA-mediated silencing of ERK3 expression in non-small cell lung cancer (NSCLC) cell lines A549 and H1299 decreased migration and invasion, while expression of exogenous ERK3 led to increased motility (Long et al., 2012; Elkhadragy et al., 2018). Furthermore, stable ERK3 knockdown in H1299 cells resulted in a significant decrease in the number and size of tumor nodules formed in the lungs of a xenograft tumor model (Long et al., 2012). Within cultured cells, ERK3 is constitutively localized in the cytoplasm and nucleus (Julien et al., 2003). Additionally, accumulation of the protein at the cell periphery is known to occur, where it orchestrates F-actin-dependent morphological changes that reduce cell spreading and adhesion to promote migration (Al-Mahdi et al., 2015).

The location, amplitude, and duration of ERK3 signaling must be carefully controlled to elicit an appropriate cellular response (Mathien et al., 2021). Phosphorylation of Ser189 in the activation loop is critical for ERK3 kinase activity (Elkhadragy et al., 2018) and is mediated, in part, by the group I p21-activated kinases (PAKs) (De la Mota-Peynado et al., 2011; Déléris et al., 2011), whereas Ser189 dephosphorylation is mediated by dual-specificity phosphatase 2 (DUSP2) (Perander et al., 2017). In addition, the regulation of ERK3 protein stability is believed to play a major role in controlling its biological activity (Coulombe et al., 2003; Mathien et al., 2017). The identification of additional ERK3-binding proteins will be invaluable for revealing the mechanisms of ERK3 regulation in normal and cancer cells and, ultimately, for deciphering ERK3 function. In this regard, DGKζ was first shown to interact with ERK3 in a yeast two-hybrid assay to identify previously unknown modulators of this extracellular signal-regulated protein kinase pathway (Vinayagam et al., 2011). Here, we verified and characterized the interaction between DGKζ and ERK3 *in vitro* and in cultured human cells. We show that DGKζ binds directly to the C34 domain of ERK3 and inhibits the ability of ERK3 to promote lung cancer cell migration.

## 2 Materials and methods

### 2.1 Antibodies

Antibodies used in this study are shown in Supplementary Table S1.

### 2.2 Cell culture and transfection

A549 and H1299 cells were cultured in RPMI, and 293T cells were cultured in DMEM. The media were supplemented with 10% fetal bovine serum (FBS) and 100 μg/mL penicillin–streptomycin. All the culture media, PBS, FBS, trypsin, and antibiotics were purchased from Gibco/ThermoFisher Scientific.

Plasmid transfections were conducted using FuGENE HD (Promega) or Lipofectamine 3000 (Invitrogen) transfection reagents, depending on the cell line. Plasmids were incubated with the transfection reagent in serum-free media and added dropwise to healthy, sub-confluent cells. For transient protein silencing, the appropriate siRNA (e.g., Qiagen’s siControl, 1027281, or siDGKζ, SI02223347) was incubated with DharmaFECT 1 (Dharmacon) in serum-free media before treating the cells with the complex dropwise. Transiently transfected cells were incubated for 48 h (unless otherwise stated) and then processed for downstream assays.

### 2.3 Plasmids

Wild-type pCMV-HA-DGKζ and the kinase-dead mutant, pCMV-HA-DGKζ KD, were previously generated (Topham et al., 1998).

The pSG5-Flag-ERK3 (WT) plasmid was generated by moving the full-length ERK3 cDNA sequence from the pSG5-HA-ERK3 plasmid (Alsaran et al., 2017) to the pSG5-Flag vector (Long et al., 2012). pSG5-Flag and pSG5-HA-ERK3 plasmids were digested with EcoRI, which linearized the pSG5-Flag and freed the EcoRI-flanked ERK3 cDNA insert. The pSG5-Flag backbone and ERK3 insert were then ligated. ERK3 cDNA was then adjusted to be in frame with the Flag tag sequence by KpnI digestion, Klenow DNA polymerase I blunting of the ends, and re-ligation. The final pSG5-Flag-ERK3 plasmid sequence was confirmed by XhoI restriction digestion and DNA sequencing.

pSG5-Flag-ERK3 (aa341-481; C34) and pSG5-Flag-ERK3 (aa482-721; C-terminus) were generated first by PCR amplification of the aa 341–481 fragment and aa 482–721 fragment using pSG5-HA-ERK3 as the template, respectively, then by Kpn I/Nhe I double digestion of PCR products and the pSG5-Flag vector, and subsequent T4 DNA ligase-mediated ligation of each ERK3 fragment into the pSG5-Flag vector. The PCR primers used for amplifying the 341–481 aa fragment are the forward primer 5′-GGGGTACCATTTTGCTTA TGG​ATG​AAA​CTC​ACA​GTC​ACA​TTT​A-3′ containing a Kpn I site and the reverse primer 5′- GGG​GCT​AGC​CTA​TTC​TTT​CCA​ATT​GGA​AAG​ATC​TAT​AAT​AAG​CTT​GG-3′ containing an Nhe I site. The PCR primers used for amplifying 482–721 aa fragment are the forward primer 5′- GGG​GTA​CCC​AAA​GCA​AAG​AAA​AAT​CTG​ATA​AGA​AAG​GCA​AAT​C-3′ containing a Kpn I site and the reverse primer 5′- GGG​GCT​AGC​TTA​GTT​CAG​ATG​TTT​CAG​AAT​GCT​GCT​GTA​TGT​TTG-3′ containing a Nhe I site.

The pSG5-Flag-ERK3 (Kinase) plasmid expressing the kinase domain of ERK3 was generated by moving the ERK3 (aa1-340; kinase) sequence from the pSG5-HA backbone to the pSG5-Flag backbone. To perform this, pSG5-HA-ERK3 (Kinase) and pSG5-Flag-ERK3 were treated with EcoRI. Dephosphorylation of the vector with CIP prevented self-ligation. After agarose gel separation, the ERK3 (Kinase) segment and the pSG5-Flag backbone segments were extracted. These two sequences were then ligated together. The final pSG5-Flag-ERK3 (Kinase) plasmid sequence was confirmed by ApaI restriction digestion analysis and DNA sequencing.

The pSG5-Flag-ERK3 (ΔC34) plasmid was generated by amplifying the kinase and C-terminal domains of ERK3 and ligating into the pSG5 backbone. pSG5-Flag-ERK3 (WT) was used as a template in PCR to amplify the kinase and C-terminal domains of ERK3 with an overlapping region. The EcoRI sites on both sides of the WT ERK3 protein sequence were included in the amplification. Primers 5’—GGA​ATT​CGG​CAG​AGA​AAT​TTG​AAA​G—3′ and 5′-CCT​TTC​TTA​TCA​GAT​TTT​TCT​TTG​CTTT​GAT​CAT​CAA​CTT​CAT​CTT​CAA​TAT​GAA​AAG-3′ were used to amplify the kinase domain, and primers 5′-CTTTTCATATTGAAGAT​GAA​GTT​GAT​GAT​CAA​AGC​AAA​GAA​AAA​TCT​GAT​AAG​AAA​GG-3′ and 5’—CGGAATTC​GCT​TAG​TTC​AGA​TGT​TTC​AGA​ATG—3′ were used to amplify the C-terminus. The pSG5-Flag-ERK3 (WT) plasmid was digested with EcoRI and the vector backbone used for the new plasmid. Purified PCR products were digested with EcoRI, subjected to EcoRI heat inactivation at 70 °C for 15 min, and then digested with BclI (a restriction digestion site unique to the kinase/C-terminus junction) for 1 h at 50 °C. The digested PCR products were ligated to the pSG5-Flag vector.

The pCMV-HA DGKζ (Δ1-233) plasmid was generated as described briefly here: an EcoRI site lies just upstream of DGKζ cDNA in the pCMV-HA DGKζ plasmid, and a HindIII site lies downstream of the codon of amino acid 234 of DGKζ. PCR was performed using primers 5′GG​AAT​TCG​GTC​CAC​GCA​GCC​GTG​GTC 3′ and 5′ GCA​AGC​TTG​CAG​GGC​TCG​CCA​TCC 3’ to amplify a product containing the EcoRI site and the nucleotide sequence covering amino acids 234 to 604 of DGKζ that includes a HindIII site. pCMV-HA DGKζ and the PCR product were then digested with EcoRI and HindIII and ligated together.

A list of previously described plasmids is summarized in Supplementary Table S2.

### 2.4 Bacterial protein isolation

His_6_-ERK3-GST (WT) was generated by expressing the pHGST.1-ERK3 plasmid (Coulombe and Meloche, 2002), and His-ERK3 (1–340; Kinase) was generated by the expression of the pET28b (+) His-ERK3(Kinase) plasmid (Elkhadragy et al., 2018) in BL-21 cells following the procedures described in Elkhadragy et al. (2018).

Glutathione S-transferase (GST) was generated by expressing pGEX-4T-1 in BL-21 cells and growing the culture under similar conditions to those of the previous protein. Upon induction with IPTG, the culture was incubated at 37°C for 6 h. Again, cells were lysed using sonication, and the supernatant was incubated with glutathione resin. The resin was washed, and the protein was eluted with 10 mM glutathione.

The GST-tagged DGKζ fragments for the N-terminus, C1, MARCKS, 1/2 kinase, 2/2 kinase, ankyrin repeats, C1A, and C1B were partially separated in bacterial lysates before being separated by size on an SDS-PAGE gel. To begin, plasmids pGEX-4T-1 (N-terminus), pGEX-4T-1 (C1), pGEX-4T-1 (MARCKS), pGEX-4T-1 (1/2 kinase), pGEX-4T-1 (2/2 kinase), pGEX-4T-1 (ankyrin repeats), pGEX-4T-1 (C1A), and pGEX-4T-1 (C1B) were expressed in BL-21 bacteria (Ard et al., 2012). Once the diluted cultures grew to an OD_600_ of about 0.6, they were induced with IPTG for 4 h at 37°C. Cells were lysed using Buffer A and sonication. The insoluble fractions were re-suspended in equal volumes of PBS and 5x loading dye and boiled. For the ankyrin repeats, which were mostly soluble, the supernatant was transferred to a new tube, and an equal amount of 5x Laemmli sample buffer was added. After boiling, the samples were stored at −20°C.

To quantify the isolated protein, the protein was mixed with 5x Laemmli sample buffer and separated from impurities on an SDS-PAGE gel. A standard curve of quantified BSA was also run in each gel for comparison to isolated protein. Coomassie staining was performed with InstantBlue Protein Stain (by Expedeon). SDS-PAGE gels were placed in a sufficient amount of the reagent to cover the gel and gently rocked at room temperature overnight. Stained gels were then washed with water for a few hours to a few days and imaged.

### 2.5 Co-immunoprecipitation

Cells were rinsed with ice-cold PBS and lysed using EBC lysis buffer (50 mM Tris (pH 7.5), 150 mM NaCl, and 0.5% NP-40) with 1 mM complete protease inhibitor cocktail (Roche) and 1 mM phosphatase inhibitor cocktail III (Sigma-Aldrich). Lysates were centrifuged, and a small sample of the cleared supernatant was set aside as “Input.” The remaining cleared supernatant was incubated with resin beads in a preclearing step. The precleared lysate was transferred to an IgG control tube and the IP tube(s) in equivalent proportions. A control antibody was added to the IgG tube, while an antibody for the protein of interest was added to the IP tube(s) before incubation. The lysate and antibody mix was then transferred to beads to allow the antibodies and beads to form a complex and facilitate precipitation of the protein–antibody complex. When using antibody-bound beads (such as anti-HA beads), the IgG control consisted of similar beads with no bound antibody to which rabbit or mouse IgG was added. The beads were washed to remove unbound proteins. Samples were boiled in Laemmli sample buffer for 5 min with occasional agitation, and Western blotting was performed.

### 2.6 Western blot

Cells in each sample were lysed with EBC lysis buffer and centrifuged. Laemmli sample buffer was added to the cleared supernatants, and the samples were boiled. The protein was then separated by SDS-PAGE and transferred to a nitrocellulose membrane for analysis. Membranes were blocked in 5% non-fat milk or 2% BSA in PBST for 30 min to 1 h. Proteins of interest were probed with appropriate primary antibodies and their corresponding secondary antibodies. Western blots were visualized by chemiluminescence (ECL by ThermoFisher).

### 2.7 *In vitro* binding assay

About 5 μL of protein A beads was added into tubes and blocked with 2% BSA in PBS for 1 h. The supernatant containing unbound BSA was removed, and 50 μL of EBC buffer was added to the beads. Up to 1 μL ERK3 antibody (Bethyl) was added to the beads and rotated for 1 h at 4°C, then centrifuged, and the excess buffer was removed. ERK3 antibody-bound beads in the tube were kept on ice for later use. During this time, ice-cold EBC buffer with protease and phosphatase inhibitors was added to a second set of tubes, each containing 5 μL of protein A beads. Then, 150 ng of purified DGKζ protein was added singly or in combination with 200 ng of purified ERK3 protein to the second tube containing beads to a total volume of 110 μL mix in the EBC buffer. The proteins were mixed, and 10 μL was taken as an input. The proteins were then rotated for 1 h at 4°C. The protein mix was transferred to the first tube containing antibody-bound beads and rotated for 1 h at 4°C. Beads were then washed three times, each for 3 min with 1 mL of EBC buffer at 4 °C. Laemmli sample buffer was added to elute the proteins bound on the beads, and the samples were boiled. Western blotting was performed to determine protein binding.

### 2.8 Immunofluorescence

Cells were plated in 24-well plates on coverslips and treated or transfected as needed. About 32–48 h after transfection, cells were fixed with 4% paraformaldehyde and washed three times with PBS. When probing with an antibody or staining with phalloidin, the cells fixed with 4% paraformaldehyde were washed three times with PBS and permeabilized with 0.15% Triton X-100 for 5 min. Cells were washed again three times with PBS and blocked with 5% goat serum or 2% BSA in PBS for 45 min. The primary antibody was then mixed with blocking solution and fixed cells were incubated with the antibody overnight at 4°C. The next day, the excess antibody was removed by washing five times with PBS, and the fluorescein-conjugated secondary antibody was added for 30 min at room temperature. Excess antibody was again removed by washing five times with PBS. Antibodies were fixed with 4% paraformaldehyde at room temperature for 30 min, and fixed cells were washed three times with PBS. Samples were stained with 4′,6-diamidino-2-phenylindole (DAPI; 5 μg/mL) in water at RT for 5 min. Cells were washed in PBS three times for 5 min and then incubated in 50 mM ammonium chloride for 15 min at room temperature to quench formaldehyde autofluorescence. Cells were again washed two times in PBS and then mounted on slides using an antifade reagent. Samples were stored at 4°C.

### 2.9 Protein–protein overlay assay

The insoluble or soluble fractions of the bacterial lysate, as well as isolated GST protein, were mixed with 5x Laemmli loading dye and were loaded twice (in two sets of wells) in an SDS-PAGE gel and then separated by size. Proteins were then transferred to a nitrocellulose membrane. The membrane was incubated with Ponceau stain and then washed with water to remove excess stain and visualize the proteins. Ponceau staining was imaged using a Galaxy S10e camera phone. Staining was removed by washing with PBST, and then the blot was blocked with 5% non-fat milk PBST. The blot was then cut, separating each series of samples. One or more sets of samples were washed with a protein of interest (e.g., bacterially derived His_6_-ERK3-GST) in PBST for 1.5 h, while the remaining series was washed in PBST alongside the others. The blots were washed and probed with an antibody against the incubated protein. After washing again, the blot was probed with the appropriate secondary antibody before the final series of washing and imaging.

### 2.10 Two-chamber Transwell^®^ migration and invasion assays

Cells were treated or transfected as necessary; for transient transfection, cells were transfected and incubated 24–48 h prior to the assay. For migration assays, 45,000 H1299 cells were then transferred to Transwell^®^ inserts by trypsinizing, counting, and re-plating cells in the upper chamber of Transwell^®^ inserts in serum-free media. Complete media (containing 10% FBS) was added in the bottom chamber. After 14.5 h, the cells on the bottom of the insert were fixed with formaldehyde, stained with crystal violet, and imaged. Images (or “fields”) were counted manually or using ImageJ and reported as the percentage of migrated “Cells/Fields.” For the invasion assay, 60,000 A549 cells were added to Transwell^®^ inserts pre-coated with 1 mg/mL growth factor-reduced Matrigel (BD Biosciences) and allowed to invade for 16 h, as was previously described (Elkhadragy et al., 2018).

### 2.11 Statistical analysis

Statistical analyses were applied when appropriate and significance (*p*-value ≤0.05) is indicated by an asterisk (*) in the figures. The statistical test(s) used for each experiment are noted in the corresponding figure legend. Student’s t-test was used to determine significance between two groups, one-way ANOVA and Tukey’s *post hoc* test or a mixed-effects ANOVA (which takes unequal variance into consideration) were used to determine significance between three or more groups, and Kruskal–Wallis test and Dunn’s *post hoc* test were used to compare the categorical trends between groups shown in Figure 7.

## 3 Results

### 3.1 ERK3 and DGKζ interact directly

**FIGURE 1:**
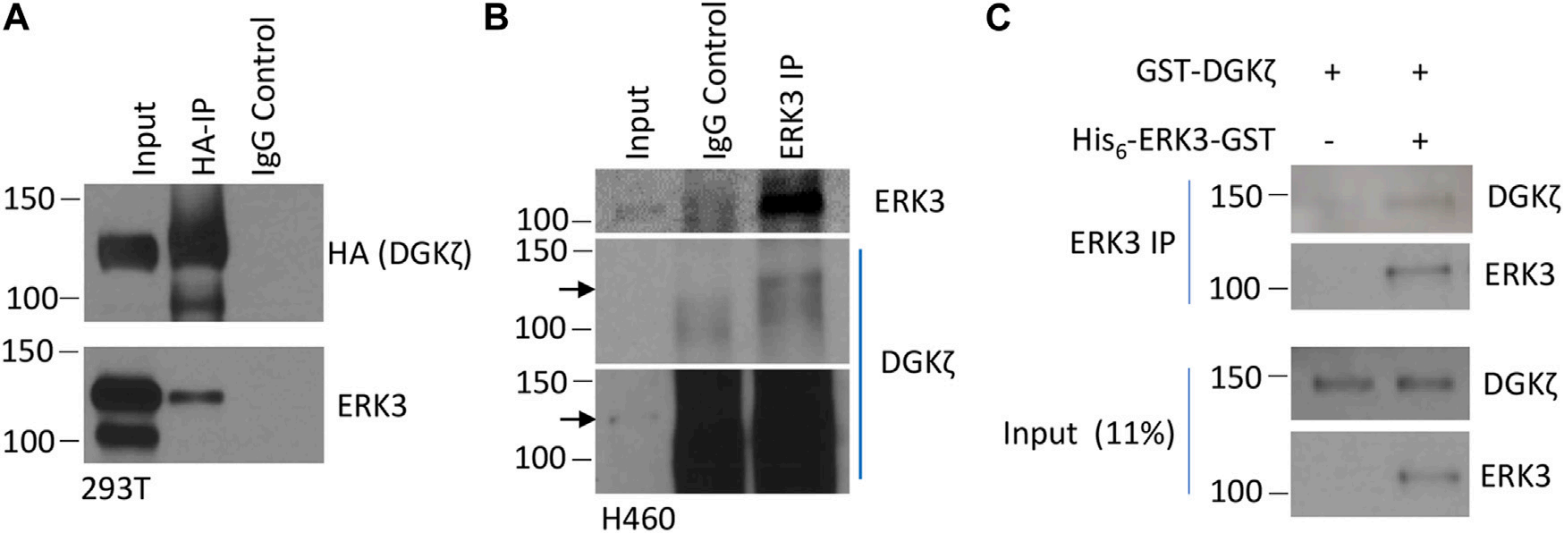
ERK3 and DGKζ interact. **(A)** Western blot analysis of the IP of HA-tagged DGKζ using either anti-HA beads (HA-IP) or mouse IgG (IgG control) to determine the presence of ERK3 in IP samples after co-overexpression of ERK3-Flag and DGKζ-HA in 293T cells. The input was 2% of the analyzed lysate for the HA blot and 4% for the ERK3 blot. **(B)** Western blot showing the IP of endogenous ERK3 in H460 cells using either anti-ERK3 antibody (ERK3 IP) or rabbit IgG (IgG control) to assess endogenous DGKζ binding. The input was 2% of the analyzed lysate. Both a shorter exposure (the upper panel) and a longer exposure (the lower panel) are shown due to high non-specific DGKζ signals in IP samples. Arrows mark the same molecular weight between the two exposures. **(C)** Western blots of the *in vitro* binding assay after IP using the ERK3 antibody of purified DGKζ incubated with (right lane) or without (left lane) purified His_6_-ERK3-GST. Numbers on the left side of the Western blots indicate molecular weight (in kDa).

To verify that ERK3 and DGKζ interact in mammalian cells, exogenous HA-tagged DGKζ and Flag-tagged ERK3 were co-expressed in human embryonic kidney 293T cells. DGKζ was immunoprecipitated (IPed) using an anti-HA antibody coupled to beads and subjected to SDS-PAGE analysis and immunoblotting to visualize the bound proteins (Figure 1A). ERK3 was captured with the complex immunoprecipitated by the anti-HA antibody, but not by control IgG, indicating its association with DGKζ. Next, the interaction of endogenous ERK3 and DGKζ was tested by co-IP in the NSCLC cell line NCI-H460 (Figure 1B). DGKζ was only observed in the ERK3 IP sample, suggesting DGKζ forms a stable complex with ERK3 in cells.

Then, we determined whether this interaction occurred directly using purified proteins in an *in vitro* binding IP assay. GST-DGKζ was purchased and the recombinant His_6_-ERK3-GST expressed in bacteria was purified (Elkhadragy et al., 2018). The purified proteins were examined by Coomassie blue staining, and their identity was confirmed by immunoblotting with an anti-ERK3 antibody (Supplementary Figures S1A, S1B). The proteins were mixed and incubated, and then ERK3 was immunoprecipitated with an anti-ERK3 antibody. The bound proteins were analyzed by SDS-PAGE and immunoblotting (Figure 1C). GST-DGKζ was captured by anti-ERK3 in the presence of His_6_-ERK3-GST, but not when ERK3 was omitted, indicating the interaction between DGKζ and ERK3 is direct.

### 3.2 ERK3 and DGKζ localize near the plasma membrane

**FIGURE 2:**
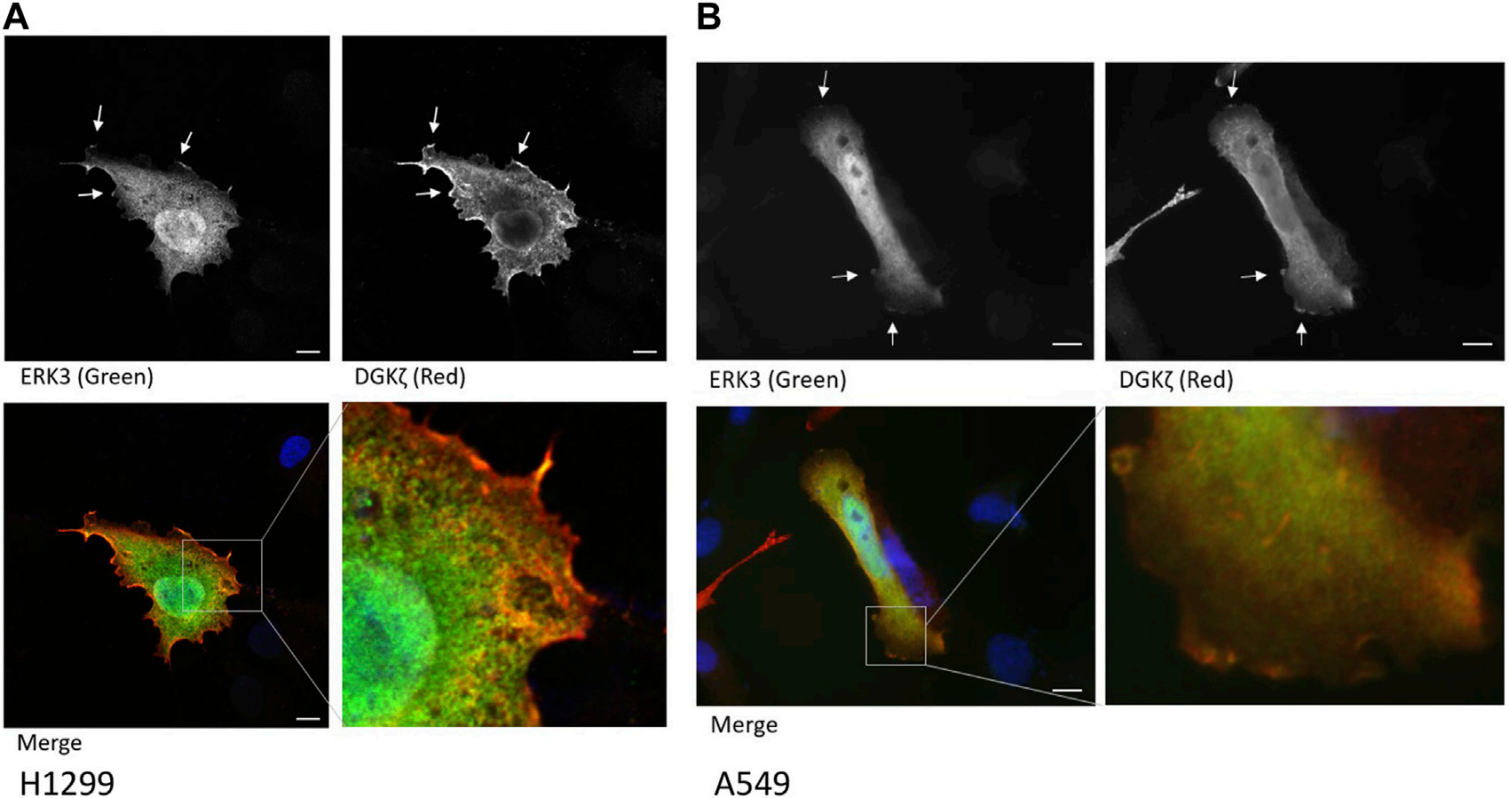
ERK3 and DGKζ co-localize at cell membrane protrusions. **(A)** Confocal microscopy images of an example cell with overexpression of Flag-ERK3 (stained with ERK3 antibody; green) and HA-DGKζ (stained with HA antibody; red) in fixed H1299 cells. Cells were imaged on an Olympus FV1000 microscope using ×60 objective. Scale bars: 10 µm. Arrows indicate the example areas of co-localization at the plasma membrane. **(B)** Fluorescent microscopy image of overexpressed Flag-ERK3 (ERK3 antibody; green) and HA-DGKζ (HA antibody; red) in fixed A549 cells. Cells were imaged on a Zeiss Observer D1 using the ×63 objective. Scale bars: 10 µm.

To determine their cellular co-localization, Flag-ERK3 and HA-DGKζ were co-expressed in H1299 NSCLC cells. Cells were stained with anti-HA and anti-Flag antibodies, followed by fluorescently labeled secondary antibodies, and with DAPI to identify nuclei and imaged by confocal microscopy. Flag-ERK3 and HA-DGKζ were co-localized at some regions near or on the plasma membrane, especially at cellular protrusions. However, the proteins did not appear to co-localize in the cytosol, seemingly confined in separate regions throughout the cells, presumably different organelles (Figure 2A, Supplementary Figures S2A, S2B). This was confirmed with a similar experiment co-overexpressing ERK3 and DGKζ in A549 cells. While this experiment was performed with a fluorescent microscope rather than a confocal fluorescent microscope, the co-localization of ERK3 and DGKζ near the cell periphery is evident (Figure 2B, Supplementary Figure S2C). As both proteins have previously been reported to be involved in cytoskeletal changes and cell motility, their interaction mainly at cell membrane regions may be indicative of an interplay between ERK3 and DGKζ in this cell process (Abramovici et al., 2009; Al-Mahdi et al., 2015).

### 3.3 The C34 domain of ERK3 is important for interaction with DGKζ

**FIGURE 3:**
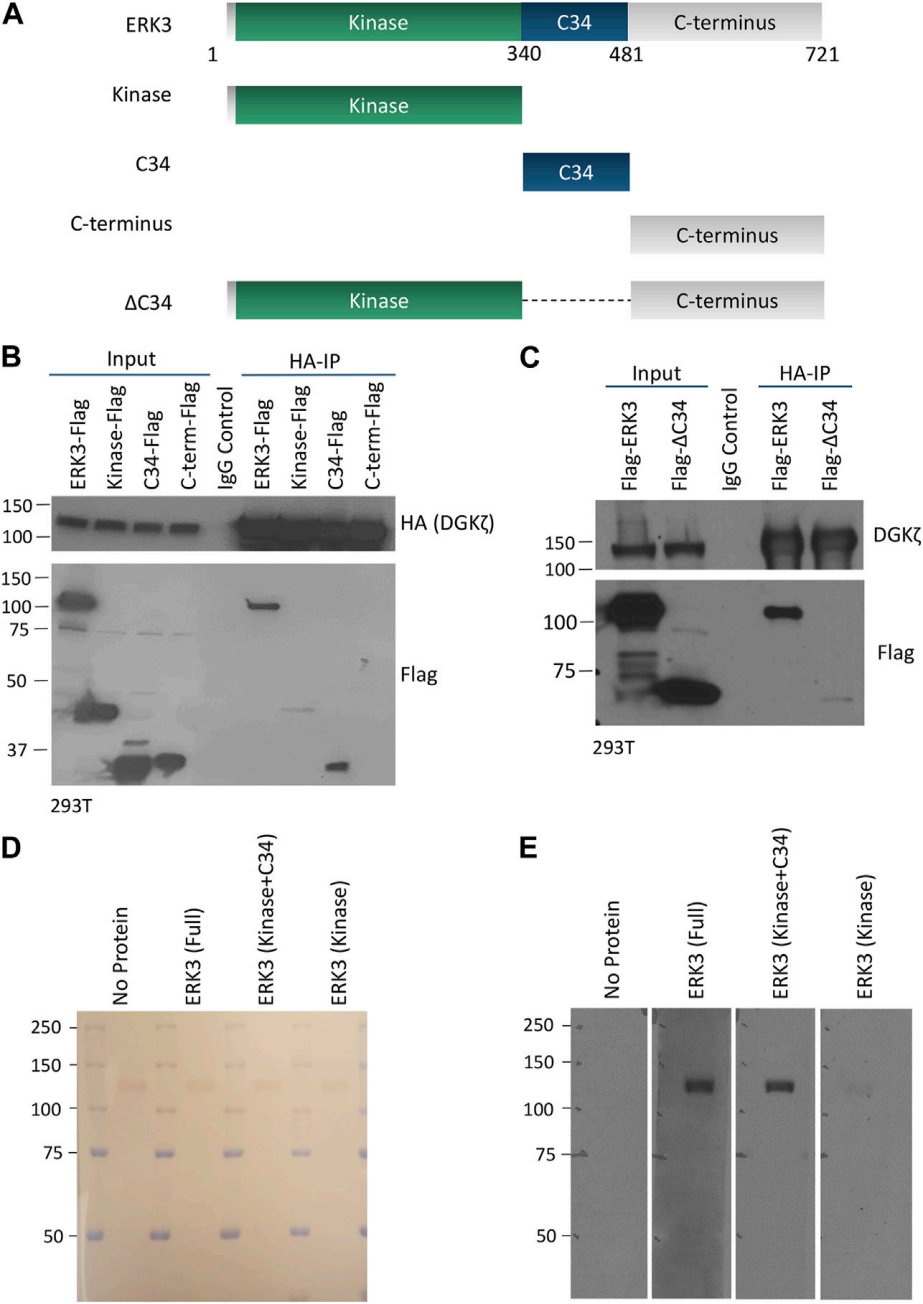
The C34 domain of ERK3 is important for interaction with DGKζ. **(A)** Schematic of ERK3 full-length protein and domains cloned for separate expression in mammalian cells. **(B)** HA-DGKζ was IPed from the 293T cell protein lysate after co-overexpression with each of the Flag-tagged ERK3 constructs as indicated. Western blot was performed using Flag antibody to assess the comparative binding strength of ERK3 domains to IPed DGKζ. Mouse IgG antibody was used to perform the IgG control and detect non-specific binding. Molecular weight is indicated by the numbers on the left side in each blot. **(C)** Flag-ERK3 or Flag-ERK3 ΔC34 were co-overexpressed with HA-DGKζ in 293T cells. HA-DGKζ was IPed using anti-HA beads. Samples were subjected to Western blotting using Flag antibody for evaluating the binding of the ΔC34 mutant against the binding of the full-length ERK3. **(D,E)** Protein–protein overlay assay in which nitrocellulose membrane blots bound with size-separated HA-DGKζ protein (visualized by Ponceau stain, D) were overlaid with PBST (no protein control), full length ERK3, the kinase + C34 domains, or the kinase domain. The binding of ERK3 domains to HA-DGKζ was then analyzed by Western blotting using the ERK3 antibody that recognizes an epitope at the N-terminus of ERK3 protein **(E)**.

To identify the protein domains in DGKζ and ERK3 that mediate their mutual interaction, we first tested a series of ERK3 truncation mutants (shown schematically in Figure 3A) for the ability to bind HA-DGKζ. Flag-tagged full-length (WT) ERK3 or one of the three ERK3 domains (kinase, C34, or C-terminal), was co-expressed with HA-DGKζ in 293T cells. DGKζ was immunoprecipitated using anti-HA beads, and immunoblotting was used to assess the relative amounts of each ERK3 domain captured compared to full-length ERK3 (Figure 3B). Only the C34 domain was efficiently co-immunoprecipitated with DGKζ. Some binding of the ERK3 kinase domain was detected, suggesting low affinity for DGKζ or indirect complexing with DGKζ under these conditions. To determine if the C34 domain is necessary for ERK3 to bind to DGKζ, we tested an ERK3 deletion mutant lacking the C34 domain (ΔC34, Figure 3A) in the same assay. Compared to full-length ERK3, the binding of ΔC34 to DGKζ was substantially reduced (Figure 3C). Collectively, these results suggest the C34 domain comprises the main binding site for DGKζ on ERK3; however, weak binding to the ΔC34 mutant and the isolated kinase domain suggest the latter may contribute to the ERK3–DGKζ interaction.

An *in vitro* protein–protein overlay assay was used to further test the requirement of the C34 domain for ERK3 interaction with DGKζ. Full-length His_6_-ERK3-GST, a His-ERK3 kinase domain, and a His-ERK3 construct containing both the kinase and C34 domains were isolated from bacteria. The purity of the bacterial-derived proteins is shown by Coomassie staining, as observed in Supplementary Figures S1B–S1D. HA-DGKζ isolated by immunoprecipitation from 293T cells was analyzed by SDS-PAGE and transferred to a nitrocellulose blot. Ponceau staining confirmed HA-DGKζ migrated at ∼120 kDa as expected, and equivalent amounts were loaded in each of the four replicate lanes (Figure 3D). The blots were overlaid with each of the purified His-ERK3 recombinant proteins, followed by an anti-ERK3 antibody and an HRP-labeled secondary antibody. A prominent band corresponding to the size of DGKζ was observed when the blots were incubated with either full-length ERK3 or the ERK3 construct containing both the kinase and C34 domain (Figure 3E). However, the kinase domain alone only bound weakly to DGKζ. As expected, no signal was detected when the overlaid recombinant protein was omitted, demonstrating the specificity of the interaction. We conclude ERK3 binds directly to DGKζ and that the C34 domain is the primary binding site, albeit with some weak interactions contributed by the kinase domain.

### 3.4 The N-terminus and C1 domains of DGKζ are important for interaction with ERK3

**FIGURE 4:**
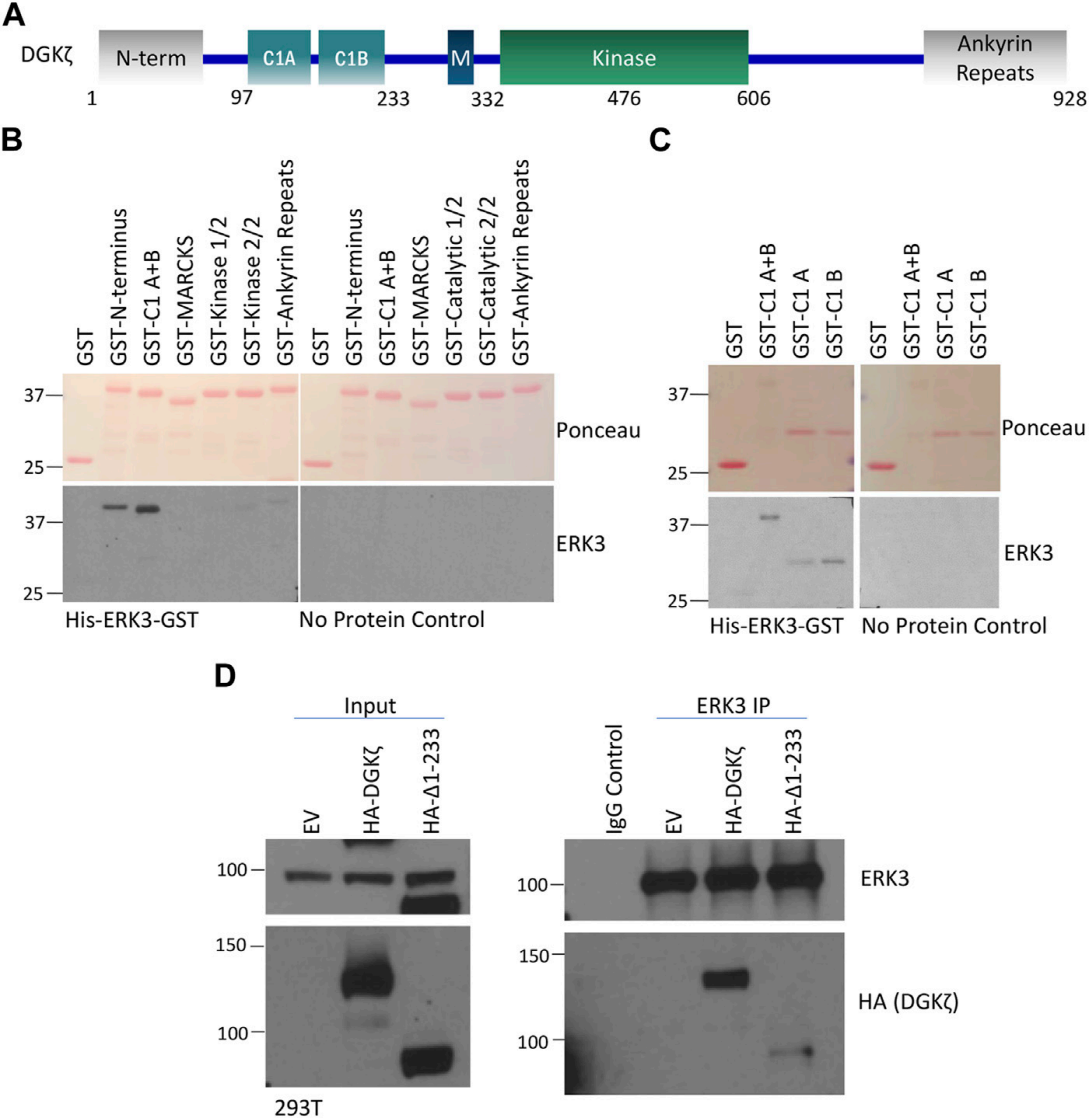
The N-terminus and C1 domains of DGKζ are important for interaction with ERK3. **(A)** Schematic of DGKζ protein domains. M, MARCKS domain. **(B)** Protein–protein overlay assay in which a blot containing partially isolated GST-tagged DGKζ deletion mutants was incubated with the His_6_-ERK3-GST protein or PBST (no protein control) and then washed and probed for bound ERK3 using an ERK3-specific antibody. The upper image shows Ponceau staining of the blot. The lower panels show Western blots probed with ERK3 antibody—the blot incubated with the ERK3 protein is on the left and the PBST (no-protein) control is on the right. GST-kinase-1/2 and GST-kinase 2/2 are the 1^st^ and 2^nd^ half of the DGKζ kinase domain with a GST tag, respectively. **(C)** A protein–protein overlay assay was performed following the same procedures as described in **(B)**, except that GST-tagged DGKζ C1 (A + B), C1A, or C1B domain mutants were assayed. Again, the blot was incubated with His-ERK3-GST or PBST for a no protein control. Isolated GST was again used as a negative control. **(D)** ERK3 IP and Western blot analysis of ERK3 protein interactions with DGKζ Δ1-233 as compared with the full-length DGKζ protein. EV, empty vector transfection control for DGKζ constructs.

To identify the domain(s) of DGKζ important for association with ERK3, a series of GST-tagged DGKζ constructs corresponding to the N-terminal, the two C1, the MARCKS-like, the first and second halves of the kinase, and the four ankyrin repeat domains were each expressed in bacteria (Figure 4A). The bacterial extracts were separated by SDS-PAGE, transferred to a nitrocellulose membrane, and then stained with Ponceau S to verify the size and relative amounts of each recombinant DGKζ protein (Figure 4B). When overlaid with purified His_6_-ERK3-GST, only the N-terminal domain and C1 domains had a strong signal. No binding was observed to other DGKζ domains or, as expected, to GST alone. Moreover, no binding was observed when recombinant ERK3 was omitted. The results suggest ERK3 binds to the N-terminal and C1 domains of DGKζ.

DGKζ contains two closely spaced C1 repeats, C1A and C1B, mediating interaction with Rac1, RhoA, and RhoGDI (which all preferentially bind to the C1A domain) (Ard et al., 2012). Therefore, a similar overlay assay was conducted to determine if a preference for ERK3 binding could be identified. The results indicated that ERK3 has some preference for the C1B repeat but also has an affinity for the C1A (Figure 4C). This is supported by the fact that the C1A + B fragment seems to have stronger binding than the individual C1A or C1B region. Thus, ERK3 may bind to DGKζ via a different mechanism than the previously studied DGKζ-C1A-binding proteins like Rac1 and RhoA.

To verify the *in vitro* results, a construct deleting the first 233 amino acids of DGKζ (HA-Δ1-233) was generated. This mutant lacks the N-terminus and C1 regions of DGKζ and may be used to ascertain whether the regions are important for ERK3 binding. A co-immunoprecipitation and Western blotting experiment was, therefore, conducted in which the full length DGKζ (HA-DGKζ) or HA-Δ1-233 was overexpressed in 293T cells, followed by immunoprecipitation of endogenous ERK3 using ERK3 antibody and Western blotting analysis of DGKζ proteins using a HA antibody (Figure 4D). Compared with the full-length HA-DGKζ, the deletion mutant HA-Δ1-233 had a remarkable reduction in binding to ERK3. This confirms that the first 233 amino acids containing the N-terminus and C1 regions are important for the DGKζ–ERK3 interaction.

### 3.5 DGKζ inhibits cell migration in NSCLC cell lines independently of kinase activity

**FIGURE 5:**
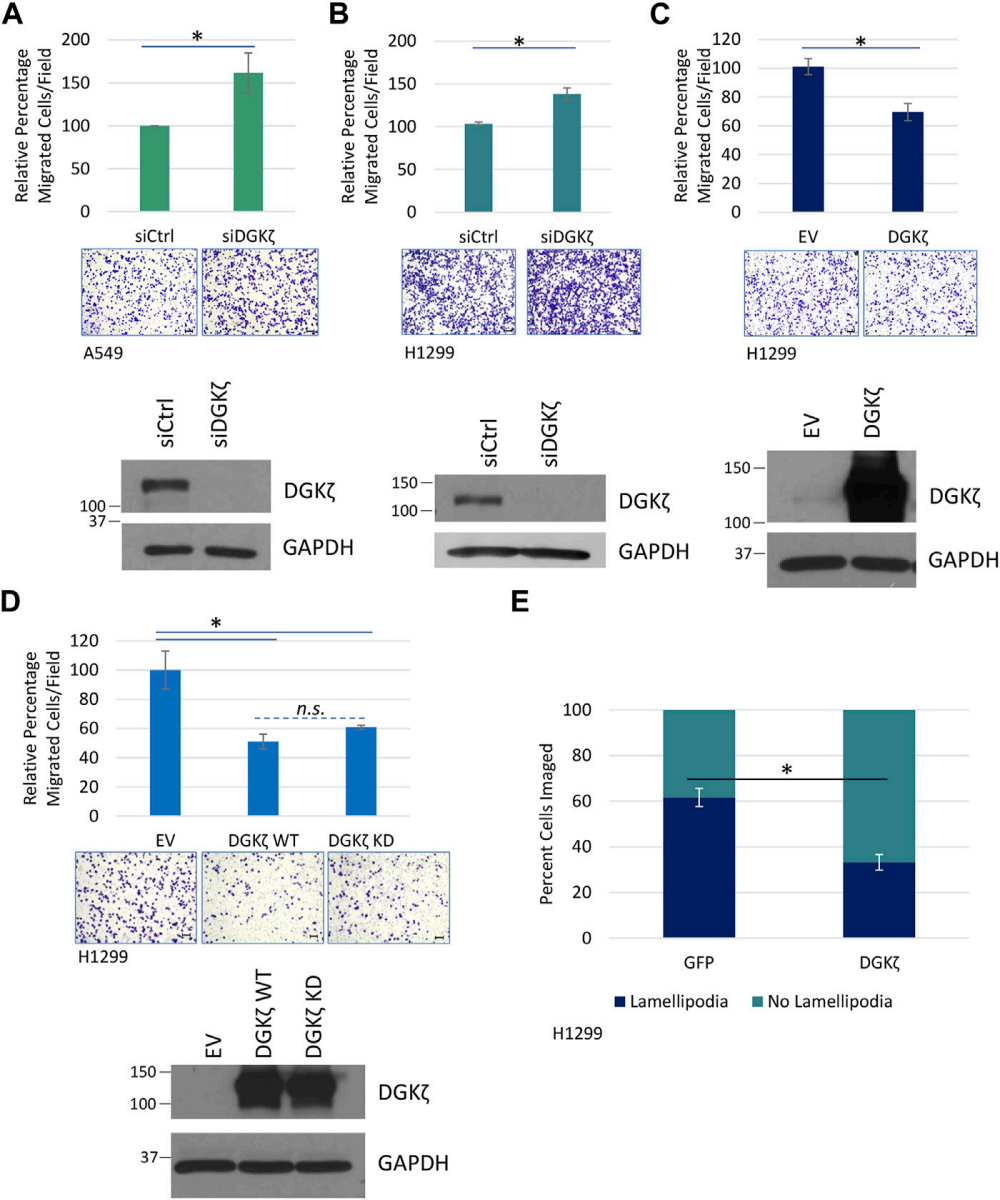
DGKζ decreases migration in NSCLC cell lines in a kinase-independent manner. **(A)** Transwell^®^ migration assay measuring cell motility after DGKζ knockdown in A549 cells. Cells transfected with siRNA targeting DGKζ (siDGKζ) or non-targeting control siRNA (siCtrl) were allowed to move through Transwell^®^ inserts for 14.5 h and then were fixed, stained, and imaged using an EVOS XL Core microscope with a ×10 objective. Values are the average of four independent experiments. Error bars represent S.E.M. Representative images of migrated cells are shown for each condition. Scale bars: 125 μm. Student’s t-test determined statistical significance. Western blotting confirmed the knockdown of DGKζ in cells transfected with siDGKζ compared to cells transfected with non-targeting siCtrl. **(B)** Transwell^®^ migration assay in H1299 cells after DGKζ knockdown as in **(A)**. Values are the average of three independent experiments. **(C)** Transwell^®^ migration in H1299 cells. Cells transfected with HA-DGKζ or the empty vector (EV) were processed as in **(A)**. Values are the average of three independent experiments. Western blotting confirmed the overexpression of DGKζ. Error bars represent S.E.M.; scale bars: 125 µm. **(D)** Transwell^®^ cell migration assay of H1299 cells transiently expressing wild-type (WT) or kinase-dead (KD) DGKζ or an empty vector (EV). Graph combined three independent experiments with bars representing S.E.M. One-way ANOVA and Tukey’s *post hoc* test were used in conjunction to assess statistical significance. n. s., not significant. **(E)** HA-DGKζ or GFP (as a non-related protein expression control) was transiently expressed in H1299 cells. After 48 h, cells were fixed, permeabilized, and visualized by immunofluorescence using an anti-HA antibody and a fluorescent secondary antibody or GFP imaging, respectively. In addition, actin was stained using phalloidin for visualizing and assessing lamellipodia structures, and chromatin was stained using DAPI to mark the nuclei. Results are the average of three independent experiments with 10–54 cells imaged depending on transfection efficiency in each experiment. Statistical significance of percent cells with lamellipodial structures between GFP and HA-DGKζ conditions were determined by Student’s t-test.

Next, we explored the cellular functions of the ERK3/DGKζ protein–protein interaction. The literature has consistently demonstrated the important role of ERK3 in cell migration and invasion (Long et al., 2012; Elkhadragy et al., 2020; Cai et al., 2021). However, there has been a discrepancy regarding the role of ERK3 in lung cancer cell proliferation. We have reported that either depletion of endogenous ERK3 or exogenous overexpression of ERK3 did not show a significant effect on cell proliferation of H1299 and A549, at least under 2D culture conditions (Long et al., 2012; Elkhadragy et al., 2018), whereas other studies reported a role for ERK3 in promoting lung cancer cell proliferation (Bogucka et al., 2021; Cai et al., 2021). Previous studies showed that DGKζ-null MEFs migrate more slowly than WT MEFs (Abramovici et al., 2009) and that increased DGKζ expression in a human metastatic colorectal cancer cell line contributed to enhanced invasiveness (Cai et al., 2014), suggesting DGKζ increases cell motility. Therefore, we focused on cell migration and sought to determine if DGKζ promotes the motility of lung cancer cells. First, the effect of siRNA-mediated silencing of DGKζ expression in A549 cells was assessed using an *in vitro* migration assay. Unexpectedly, silencing DGKζ resulted in an approximate 50% increase in the number of cells that migrated through the Transwell^®^ inserts under these conditions (Figure 5A). Similar results were obtained when DGKζ expression was silenced in the H1299 NSCLC cell line (Figure 5B). Western blotting confirmed that DGKζ expression was efficiently silenced in each cell line. These results suggest DGKζ decreases cell motility in NSCLC cells. To confirm this, the effect of increased DGKζ in H1299 cells was assessed using the same assay. DGKζ overexpression, as confirmed by Western blotting of cell lysates, resulted in an approximate 30% decrease in the number of cells that migrated through the Transwell^®^ inserts (Figure 5C), consistent with the idea that DGKζ inhibits cell migration in NSCLC cells *in vitro*.

We next investigated whether DGKζ catalytic activity is required for inhibition of NSCLC cell migration. Exogenous expression of WT DGKζ or a catalytically inactive (kinase-dead) DGKζ mutant (DGKζ KD) decreased H1299 cell migration to a comparable extent (Figure 5D). Western blotting confirmed that the two DGKζ constructs were expressed at equivalent levels. These data suggest DGKζ catalytic activity is not required for inhibition of NSCLC cell migration.

To begin to investigate the mechanistic basis for the inhibitory role of DGKζ in cell motility, we quantified lamellipodia-like protrusions in cells overexpressing DGKζ or GFP (as a control). The cells were fixed and stained with Alexa Fluor 488-conjugated phalloidin to reveal F-actin, and the number of cells with lamellipodia was quantified and expressed as a percentage of total cells. While ∼62% of GFP-expressing control cells had formed protrusions, only ∼33% of DGKζ-expressing cells exhibited these structures (Figure 5E), suggesting DGKζ overexpression inhibits lamellipodia formation.

### 3.6 DGKζ affects the ability of ERK3 to promote motility through the C34 domain of ERK3

**FIGURE 6:**
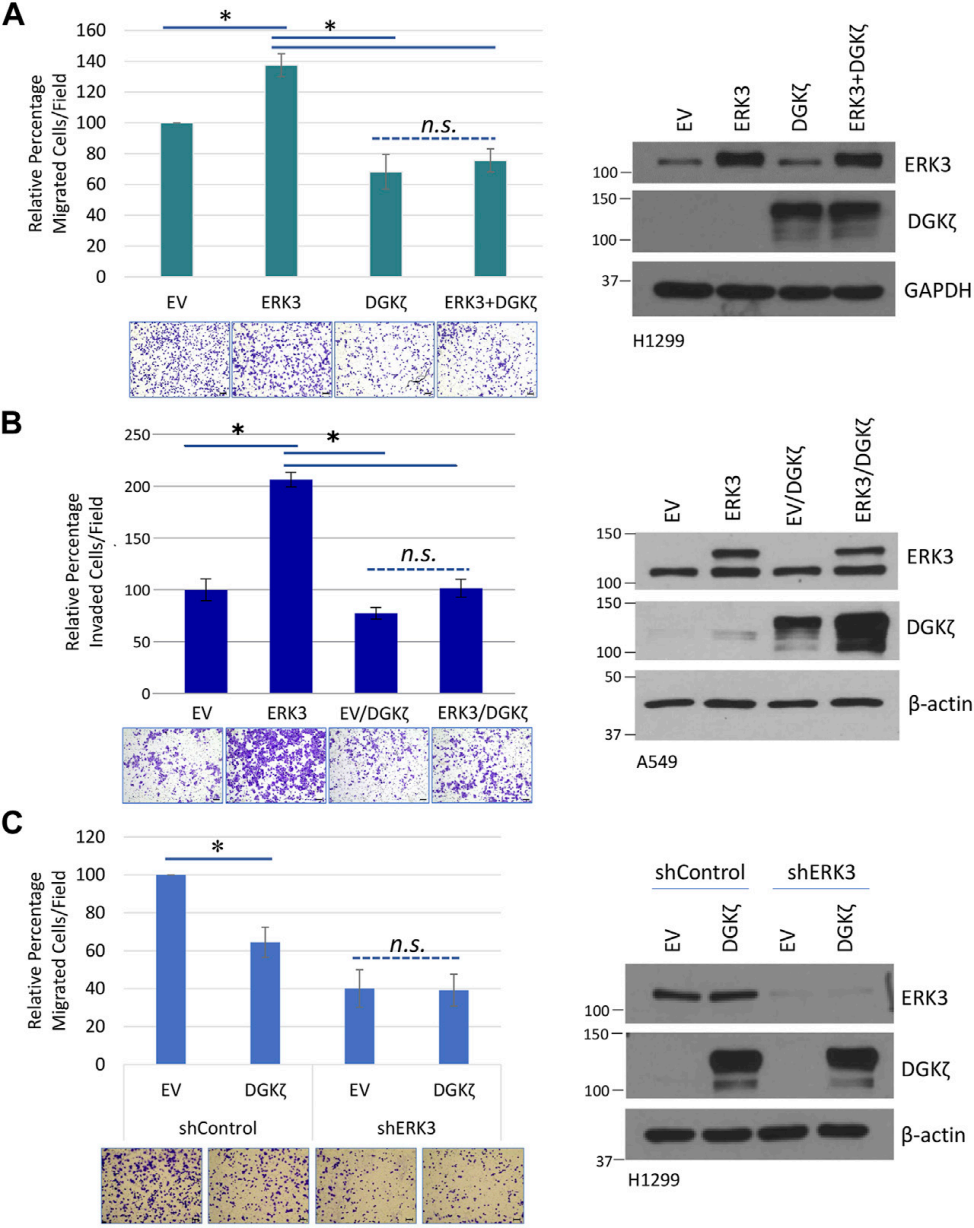
DGKζ inhibits cell migration through ERK3. **(A)** Transwell^®^ migration assay in H1299 cells with transient expression of the control empty vector (EV), DGKζ alone, ERK3 alone, or both ERK3 and DGKζ proteins. Migrated cells were imaged using an EVOS XL Core microscope with a ×10 objective. Error bars represent S.E.M. Scale bars: 125 μm. Values in graph represent the average of five independent experiments. To account for the unequal variance, a mixed-effects ANOVA was performed to assess for statistical significance. As shown on the right, a representative Western blot confirmed the overexpression of DGKζ, ERK3, or both for each condition of Transwell^®^ migration assays. **(B)** Transwell^®^ invasion assay in A549 cell lines with stable expression of an empty vector (EV) or ERK3. Both EV and ERK3 stable cells were transiently transfected with DGKζ plasmid or a control plasmid (not indicated in the figure). Invaded cells were imaged using an EVOS XL Core microscope with a ×10 objective. Values represent three biological replicates and are representative of two independent experiments. One-way ANOVA and Tukey’s *post hoc* test were used to determine statistical significance. Western blots on the left indicated the expression levels of ERK3 (lower band: endogenous ERK3; upper band: exogenous Flag-tagged ERK3) and DGKζ. **(C)** Transwell^®^ migration assay in which a control empty vector (EV) or DGKζ plasmid was overexpressed in H1299 cells, expressing either a non-targeting shRNA control (shControl) or H1299 cells with ERK3 stable knockdown (expressing shERK3). Migrated cells were imaged using an EVOS XL Core microscope with a ×10 objective. Values in graphs represent the mean of four independent experiments. Student’s t-test was used to compare statistical significance between the EV and DGKζ overexpression in shControl and shERK3 cells. As shown on the right, a Western blot confirmed the knockdown of ERK3 and overexpression of DGKζ in each experiment.

Given their opposing roles in cell motility, we next determined whether DGKζ could counteract ERK3-mediated promotion of lung cancer cell migration. HA-DGKζ and Flag-ERK3 were expressed either alone or in combination in H1299 cells. As expected, Flag-ERK3 expression by itself increased the number of H1299 cells that migrated through the Transwell^®^ inserts by approximately 40% compared to the control, an empty vector, and HA-DGKζ expression decreased cell migration by approximately the same amount (Figure 6A). However, Flag-ERK3 failed to increase cell migration when co-expressed with HA-DGKζ, despite equivalent expression to the ERK3-alone condition. Moreover, there was no significant difference in the percentage of migrated cells when DGKζ was expressed alone and when DGKζ was co-expressed with ERK3. This effect was further explored in an invasion assay using A549 cell lines stably overexpressing ERK3 or an empty vector (EV) control. HA-DGKζ or a corresponding empty plasmid was transiently overexpressed in each cell line and the ability of cells to invade was compared. The results were similar to those of the migration assay: while ERK3 significantly increased cell invasion, DGKζ co-overexpression abolished this effect, and when expressed alone, DGKζ mildly decreased the ability of cells to invade (Figure 6B). Collectively, these results suggest DGKζ expression is sufficient to inhibit lung cancer cell migration and invasion.

Due to the inverse effect on cell migration and invasion, we tried to determine if there was a correlation, inverse or otherwise, in the expression of ERK3 and DGKζ in clinical samples of lung cancer. Data analyzed using the GEPIA2 web server indicate that while ERK3 mRNA (as MAPK6 gene expression) is increased in tumor samples, reaching statistical significance in lung squamous cell carcinoma, DGKζ (as DGKZ gene expression) has a non-statistically significant decrease in the same samples (Supplementary Figures S3A, S3B) (Tang et al., 2017). This is supported by correlation data, which suggests a slightly inverse relationship between the two mRNA expression levels (Supplementary Figures S3C, SD) (Cerami et al., 2012; Gao et al., 2013).

To determine if DGKζ requires endogenous ERK3 to exert its inhibitory effect on cell migration, HA-DGKζ was expressed in H1299 cells that stably express either an shRNA targeting the 3′-UTR non-coding region of ERK3 (shERK3) or a non-targeting shRNA control (shControl) (Figure 6C). Western blotting showed ERK3 expression was efficiently silenced by shERK3, but not by shControl. While the shControl H1299 cells showed the expected decrease in migration after HA-DGKζ expression, the shERK3 cells showed no significant change after HA-DGKζ expression, despite the HA-DGKζ levels being equivalent in both conditions. These results suggest DGKζ-mediated inhibition of lung cancer cell migration requires endogenous ERK3 and inhibits ERK3-dependent cell motility pathways.

Our previous work showed that phosphorylation of Ser189 in the ERK3 activation loop is important for its ability to promote cancer cell invasiveness (Elkhadragy et al., 2018). To determine if DGKζ-mediated inhibition of lung cell migration involves changes in the levels of phosphorylated Ser189 (pSer189), an antibody specific to pSer189 was used to probe the ERK3 Ser189 phosphorylation status (Supplementary Figure S4). SiRNA-mediated silencing of DGKζ in H1299 cells followed by immunoprecipitation of total ERK3 from the cell lysates showed no detectable difference in pSer189 levels from control siRNA-treated cells. In a separate experiment, cells expressing increasing amounts of HA-DGKζ showed no changes in pSer189 levels. Collectively, these results suggest DGKζ expression does not affect the phosphorylation status of Ser189, and hence ERK3 activation.

**FIGURE 7:**
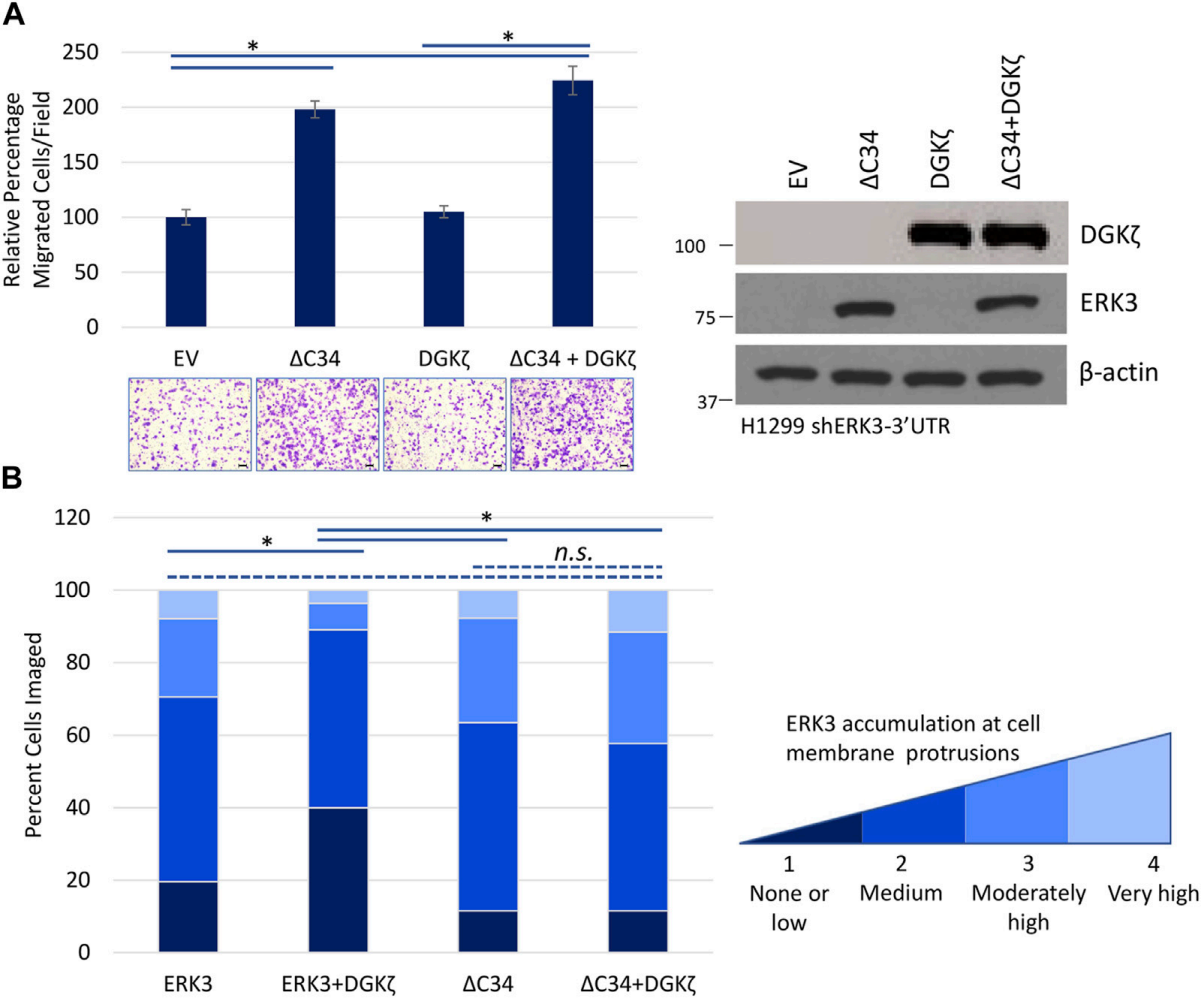
The C34 domain of ERK3 is required for DGKζ to influence ERK3-mediated cell migration. **(A)** Transwell^®^ migration assay in which ERK3 ΔC34, DGKζ, or both proteins were overexpressed in H1299 shERK3-3′UTR cells. Migrated cells were imaged using an EVOS XL Core microscope with a ×10 objective. One-way ANOVA and Tukey’s *post hoc* test were used to determine statistical significance using six fields per condition. Values in the graph are representative of four biological replicates from two independent experiments. On the right is a Western blot confirming overexpression of ERK3 ΔC34 and DGKζ. **(B)** Flag-ERK3, Flag-ERK3 + HA-DGKζ, Flag-ERK3-ΔC34, or Flag-ERK3-ΔC34 + HA-DGKζ were overexpressed in H1299 cells with an empty vector added as necessary to reach equivalent plasmid transfection for each condition. After 48 h, cells were fixed and immunostained for ERK3 or ΔC34 using a Flag antibody and for DGKζ using a HA antibody. Accumulation of ERK3 and ΔC34 at cell membrane protrusions was assessed visually using a score between 1 and 4, from “none or low” to “very high” accumulation at cell membrane protrusions. The criteria for scoring are detailed in Supplementary Table S3 with examples of cells. For each condition, >50 cells were imaged. Statistical significance was determined by the Kruskal–Wallis test and Dunn *post hoc* test.

DGKζ mainly interacts with the ERK3 C34 domain and only poorly interacts with an ERK3 mutant missing the C34 domain (ERK3ΔC34) (Figure 3). We, therefore, investigated whether DGKζ affects migration induced by Flag-ERK3ΔC34 expression. For these experiments, H1299 cells with ERK3 stably silenced were used to eliminate the possible confounding effects of endogenous ERK3. ERK3ΔC34 expression significantly increased migration through the Transwell^®^ inserts compared to the empty vector (Figure 7A). As observed in Figure 6C, migration was not affected by HA-DGKζ expression when endogenous ERK3 was silenced. Moreover, DGKζ expression had no significant effect on ERK3ΔC34-mediated cell migration, suggesting DGKζ inhibits ERK3-mediated cell migration through direct binding of the ERK3 C34 domain.

We next investigated whether the inhibitory effect of HA-DGKζ involves changes to ERK3 subcellular localization using immunofluorescence microscopy of Flag-tagged exogenous ERK3. The subcellular localization of Flag-ERK3 varied in individual cells, from strong nuclear accumulation to diffuse distribution throughout the cytoplasm. Flag-ERK3 also accumulated at cell edges or at the tips of cellular protrusions, including broad lamellipodia-like structures. To target possible changes in ERK3 localization at the cell periphery, specifically at cell membrane protrusions, Flag-ERK3 distribution was scored in individual cells, as described in Experimental Procedures and Supplementary Table S3, and the percentage of cells with low, medium, moderately high, or very high ERK3 accumulation at membrane protrusions was quantified. Compared to the expression of ERK3 alone, co-expression with HA-DGKζ resulted in fewer cells with moderately high to very high ERK3 accumulation at membrane protrusions (Figure 7B). The Flag-tagged ERK3ΔC34 mutant accumulated at protrusions to a similar extent to Flag-ERK3. However, there was no significant change in the localization of the ERK3ΔC34 when it was co-expressed with HA-DGKζ. Collectively, these results suggest ERK3 localization to membrane protrusions does not require the C34 domain but that DGKζ reduces ERK3 localization to protrusions via C34-dependent interactions.

## 4 Discussion

### 4.1 Domains that mediate the interaction of DGKζ and ERK3

Accumulating evidence points to key roles for ERK3 in the regulation of cancer cell migration and tumor metastasis, but little is known about how ERK3 activity is regulated in normal and cancer cells. In this study, we demonstrated that the lipid kinase DGKζ binds directly to ERK3 and that the two proteins form a stable complex in mammalian cells. Furthermore, DGKζ inhibits the pro-migratory effects of ERK3 in lung cancer cells, possibly by preventing ERK3 accumulation at the leading edge of cellular protrusions. Our findings add to the growing list of ERK3-interacting proteins and begin to delineate how ERK3 stimulates lung cancer cell migration.

This work shows that the ERK3 C34 domain binds directly to the N-terminal and C1 domains of DGKζ. DGKζ contains two neighboring C1 repeats, C1A and C1B, which function as protein interaction domains. GTPases Rac1 and RhoA and their mutual inhibitor, RhoGDI, bind preferentially to the C1A domain (Ard et al., 2012). To date, the only protein reported to bind the DGKζ C1B site is β-arrestin (Nelson et al., 2007). Here, we found that ERK3 also binds preferentially to the C1B domain. However, some binding to the C1A domain was observed, raising the possibility that ERK3 is capable of binding either region, potentially interacting with a sequence that is present, in part or in full, in both C1 repeats.

ERK3 also bound the N-terminal domain of DGKζ, albeit to a lesser extent than to the C1 domains. A model of the predicted three-dimensional (3D) structure of human DGKζ from the AlphaFold Protein Structure Database shows that the N-terminal region is unstructured (Jumper et al., 2021; Varadi et al., 2022). Although the AlphaFold model confidence level is low for this region, the N-terminal region may lie in close proximity to, and wraps around, the C1 domains, consistent with the possibility that ERK3 interacts with both domains, or even that ERK3 binding induces structural changes in the N-terminal domain, that then contribute to enhanced binding (i.e., an induced fit). Further experiments will be required to verify this idea. Nevertheless, our findings suggest the C1B domain and the N-terminus contribute to the stable interaction of DGKζ with ERK3.

Several lines of evidence presented here show that the C34 domain is the major binding site for DGKζ. Only recently have studies begun to shed light on the role of this domain in ERK3 function, revealing it as a key site of protein–protein interactions. One study showed that the C34 domain mediates ERK3 interaction with, and activation of, AKT (Cai et al., 2021). Another study identified the C34 domain as the main site for ERK3 binding to FBW7, a member of the F-box protein family and part of the Skp1–Cdc53/Cullin–F-box-protein (SCF) complex, which forms an E3-ubiquitin ligase that ubiquinates proteins and triggers proteasome degradation. Thus, the C34 domain contributes to the regulation of ERK3 protein ubiquitination and turnover (Walters, 2021; An et al., 2022). In contrast, the interaction of the kinase domain with the deubiquitinating enzyme USP20 regulates ERK3 protein stability, contributing to the accumulation of ERK3 protein (Mathien et al., 2017). Taken together, the current body of work suggests that the C34 domain functions as a negative regulator of ERK3 function. Given that the C34 domain is conserved in ERK3 and ERK4, one would wonder whether the C34 domain in ERK4 plays similar regulatory roles. Intriguingly, while ERK4 was also shown to interact with and activate Akt, a region in the kinase domain, instead of the C34 domain, is required for ERK4’s interaction with Akt (Wang et al., 2019). It remains to be explored whether or not the C34 domain mediates an interaction of ERK4 with DGKζ and/or FBW7.

### 4.2 How DGKζ inhibits ERK3-mediated migration

An important remaining question is how does DGKζ binding to the C34 domain reduce the localization of ERK3 at cell membrane protrusions and inhibit ERK3-mediated cell motility? Our initial hypothesis was that DGKζ inhibits phosphorylation of Ser189 within the ERK3 activation motif, which is important for localization to cell membranes and the ability to promote cell migration (Al-Mahdi et al., 2015; Elkhadragy et al., 2018). However, neither DGKζ knockdown nor overexpression altered Ser189 phosphorylation (Supplementary Figure S4). Another possibility was that DGKζ promotes ERK3 protein turnover by helping target it for degradation by the proteasome or by interfering with proteins that increase ERK3 stability (Mathien et al., 2017; An et al., 2022). However, ERK3 protein levels were not altered by DGKζ silencing or overexpression, giving less credence to this idea. Instead, we favor the idea that, via direct binding to the ERK3 C34 domain, DGKζ inhibits the retention of ERK3 on membrane protrusions and perhaps inhibits or limits interaction with motility-driving proteins, thereby decreasing ERK3-mediated cell migration. This is based on a series of findings. First, DGKζ and ERK3 co-localize primarily at cell membrane protrusions, suggesting these are the primary regions where they interact. Second, DGKζ co-expression decreased ERK3 accumulation at membrane protrusions and inhibited ERK3-mediated cell migration. Third, DGKζ had no effect on ERK3ΔC34 accumulation at membrane protrusions and ERK3ΔC34-mediated cell migration, likely due to DGKζ being incapable of binding sufficiently to ERK3ΔC34. Of note, inhibition of NSCLC cell migration by DGKζ does not require its catalytic activity, suggesting this is a kinase-independent role. One of the few other known kinase-independent roles of DGKζ is RhoA activation, which depends on the scaffolding ability of DGKζ to bring PKCα into close proximity with RhoGDI for phosphorylation and subsequent RhoA release from RhoGDI (Ard et al., 2012). Interestingly, a recent paper reported that ERK3 acted as a guanine-exchange factor (GEF) for activation of Cdc42 and Rac1 GTPases (Bogucka-Janczi et al., 2023). Perhaps, a similar mechanism is used by DGKζ to inhibit ERK3 from activating Cdc42/Rac1 and promoting cell motility.

How ERK3 becomes localized to membrane protrusions of motile cells remains a mystery. Multiple mechanisms might be involved because ERK3 promotes cell motility in both kinase-dependent and kinase-independent manners (Elkhadragy et al., 2018; 2020). The identification of membrane-targeting proteins for ERK3 would help elucidate the molecular mechanisms underlying the role of ERK3 in promoting cell motility. One intriguing possibility is that group I PAKs might mediate ERK3 localization to the leading edge of membrane protrusions, given that group I PAKs were shown to interact with ERK3 and phosphorylate ERK3 at Ser189 (De la Mota-Peynado et al., 2011; Déléris et al., 2011). It is noteworthy that PAKs’ localization to membrane protrusions requires the transport facilitated by other proteins such as Nck and β-PIX (Parrini, 2012). Therefore, it would be interesting to know whether ERK3 localization to membrane protrusions is dependent on Nck and/or β-PIX**.**

### 4.3 Contrasting roles for DGKζ in cell motility

In contrast to previous studies that show DGKζ stimulates migration in mouse embryonic fibroblasts (Abramovici et al., 2009) and colon cancer cells (Cai et al., 2014), the findings presented here show that DGKζ inhibits migration of NSCLC cells. These opposing effects might be due to cell context-dependent differences in the levels of signaling proteins, most notably of ERK3 itself. In support of this idea, cells with silenced ERK3 expression were refractory to the inhibition of migration by exogenous DGKζ expression. The overall effect of DGKζ expression on cell migration might also depend on the balance between Rac1 and RhoA signaling, both of which are critical Rho GTPases in regulating cell morphology and motility (Sadok and Marshall, 2014; Nguyen et al., 2018). DGKζ is known to activate Rac1 and RhoA in a kinase-dependent and kinase-independent manner, respectively. Interestingly, our present work shows that the effect of DGKζ on cell migration in NSCLC cell lines is through a kinase-independent mechanism (Figure 5). RhoA activation was shown to depend on the scaffolding ability of DGKζ to bring PKCα into the proximity of RhoGDI for phosphorylation and the subsequent release from RhoGDI (Ard et al., 2012). While both Rac1 and RhoA have been shown to promote cell motility in multiple cell types (Yoshioka et al., 1999; Kamai et al., 2003; Oleinik et al., 2014), Rac1 and RhoA coordinate to promote efficient movement, and dysregulation of this balance may alter cell motility (O’Connor and Chen, 2013; Nguyen et al., 2018). For example, one study in A549 cells found overexpression of constitutively active RhoA inhibited migration in a wound healing assay with significantly larger focal adhesions and indirect inhibition of Rac1 activity (Al Haddad et al., 2020). One intriguing possibility, although purely speculative, is that the presence of ERK3 at membrane protrusions is important for maintaining the balance of Rac1 signaling and RhoA signaling. Hence, in the current work, DGKζ may bind ERK3 and decrease its localization at membrane protrusions, leading to hyperactivation of RhoA and inhibition of Rac1 activity, which ultimately results in decrease in cell migration.

Activating KRAS mutations or EGFR mutations occur in a subset of NSCLCs. ERK3 expression levels are upregulated in NSCLCs, regardless of the status (wild-type or mutation) of EGFR or KRAS genes (Long et al., 2012; Bogucka et al., 2021). ERK3 promotes migration and invasion and tumorigenesis of lung cancer cells expressing either wild-type KRAS (e.g., H1299) or mutants (e.g., A549 and H23) (Long et al., 2012; Bogucka et al., 2021; Cai et al., 2021). Surprisingly, ERK3 depletion showed little effect on growth of H1650, a LUAD cell line expressing an EGFR mutant (Bogucka et al., 2021). In addition, ERK3 expression and phosphorylation at S189 were shown to be increased by either EGF stimulation (Bogucka-Janczi et al., 2023) or overexpression of constitutively active KRas mutants (Bogucka et al., 2021). Thus, it would be interesting to investigate in future work whether the DGKζ/ERK3 axis plays differential roles in migration, invasiveness, and tumor progression of NSCLCs with different statuses of KRAS or EGFR.

Additional studies are certainly needed to fully characterize the molecular actions of ERK3 on cell motility. This work has identified a novel interaction with DGKζ and a potential for ERK3 inhibition. Moving forward, a more thorough understanding of ERK3 regulation might provide novel approaches for the development of selective pharmacological tools and therapeutics to modulate ERK3 function.

## Data Availability

The original contributions presented in the study are included in the article/Supplementary Material; further inquiries can be directed to the corresponding author.
