# Supplementary material for: ERK3 and DGKζ interact to modulate cell motility in lung cancer cells
Source: Front Cell Dev Biol. 2023 May 23;11:1192221. doi: 10.3389/fcell.2023.1192221 (PMC10242005; doi:10.3389/fcell.2023.1192221)
Supplement: Supplementary file 1 [file DataSheet1.docx]

Supplementary Material

ERK3 and DGKζ interact to modulate cell motility in lung cancer cells

Amanda K Myers^1^, Marion Morel^1^, Stephen H Gee^2,3^, Katherine A Hoffmann^1^, Weiwen Long^1*^

*** Correspondence:** Weiwen Long: [weiwen.long@wright.edu](mailto:weiwen.long@wright.edu)

# Supplementary Data

## Table S1. Antibodies.

|  |  |  |  |  |
| --- | --- | --- | --- | --- |
| **Antibody (anti-)** | **Supplier** | **Catalog Number** | **Assay** | **Dilution** |
| ERK3 | Abcam | ab53277 | WB | 1:4000 |
|  |  |  | Protein overlay | 1:1000 |
|  |  |  | IF | 1:300 |
| ERK3 | Cell Signaling | #4067 | WB | 1:2000 |
|  |  |  | Protein overlay | 1:1000 |
| ERK3 | Bethyl | A302-654A | IP | 2 µg/10 cm plate |
| ERK3, p-S189 | *internally sourced* |  | WB | 1:100 |
| DGKζ | Bethyl | A362-967A | WB | 1:1000 - 1:4000 |
| GAPDH | Cell Signaling | #5174 | WB | 1:200 000 |
| β-actin | Sigma | A5316 | WB | 1:200 000 |
| Flag | Sigma | F1804 | WB | 1:5000 |
|  |  |  | IF | 1:400 |
| Flag, HRP-cojugated | Bio-Rad | 170-6516 | WB | 1:5000 |
| HA | Cell Signaling | #3724 | WB | 1:5000 |
|  |  |  | IF | 1:800 |
| HA | Sigma | H3663 | WB | 1:1000 |
| Mouse, HRP-conjugated | Bio-Rad | 170-6516 | WB | 1:5000 |
| Rabbit, HRP-conjugated | Bio-Rad | 170-6515 | WB | 1:5000 |
| Rabbit, HRP-conjugated, conformation-specific | Cell Signaling | #5127 | WB | 1:5000 |
| Rabbit, igG | Sigma | I8140 | IP | Equivalent to primary antibody of interest |
| Mouse, IgG | Sigma | 12-371 | IP | Equivalent to primary antibody of interest |
| Rabbit, Alexa Fluor 488 | Invitrogen | A11034 | IF | 1:1000 |
| Mouse, Alexa Fluor 594 | Invitrogen | A11032 | IF | 1:1000 |

## Table S2. Plasmids.

| pCMV-HA-DGKζ, WT | Mammalian expression plasmid of DGKζ (Topham et al., 1998) |
| --- | --- |
| pCMV-HA-DGKζKD | Mammalian expression plasmid of the kinase dead mutant of DGKζ (Topham et al., 1998) |
| pSG5-Flag-ERK3 (WT) | Mammalian expression plasmid of the full length ERK3 cDNA |
| pSG5-Flag-ERK3 (aa 1-340; Kinase) | Mammalian expression plasmid of ERK3 kinase domain (aa 1-340) |
| pSG5-Flag-ERK3 (aa 341-481; C34) | Mammalian expression plasmid of ERK3 C34 domain (aa 341-481) |
| pSG5-Flag-ERK3 (aa 482-721; C-terminus) | Mammalian expression plasmid of ERK3 C-terminus (aa 482-721) |
| pSG5-Flag-ERK3 (ΔC34) | Mammalian expression plasmid of ERK3 with the deletion of C34 domain |
| pCMV-HA DGKζ (Δ1-233) | Mammalian expression plasmid of DGKζ with the deletion of N-terminus (aa 1-233) |

## Figure S1. Establishment of the identity and purity of isolated proteins. (A) GST-DGKζ generated from a wheat germ system was confirmed in our laboratory by Coomassie staining and Western blotting after purchase. Molecular weight is indicated in kDa on the left side of each Coomassie Blue stained gel and Western blot. (B) His_6_-ERK3-GST generated from bacteria and quantified by Coomassie stain was confirmed as the correct protein using ERK3 antibody in a Western blot. (C) Coomassie stain analysis of His_6_-ERK3(Kinase+C34), generated from bacteria and isolated using Nickel beads. (D) Coomassie stain analysis of His_6_-ERK3(Kinase), also generated from bacteria and isolated with Nickel beads.


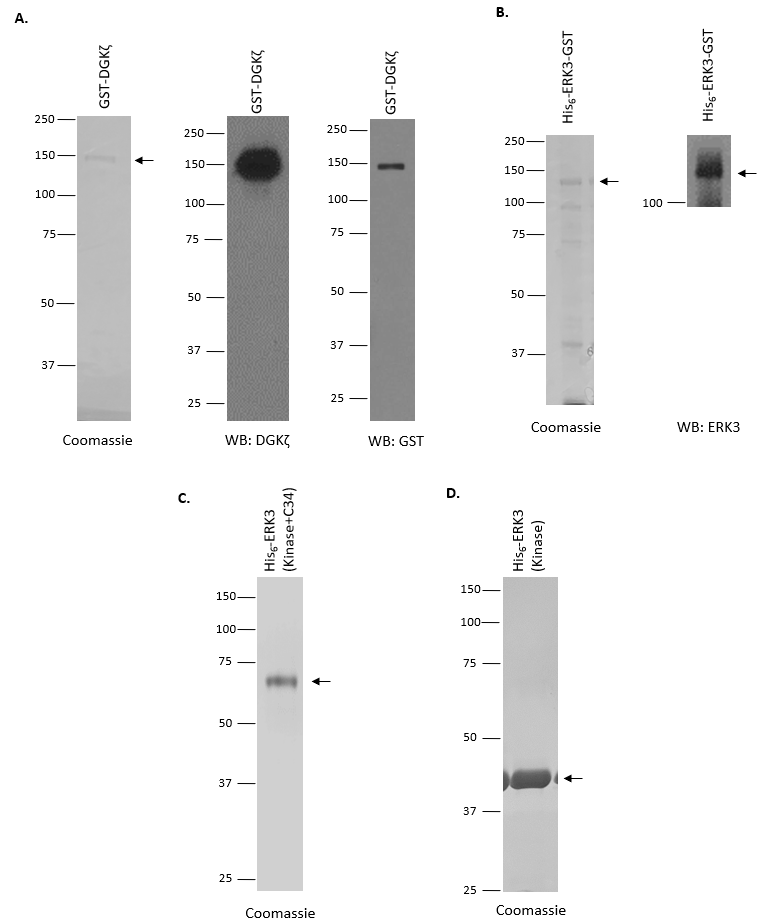


## Figure S2. Additional examples of ERK3 and DGKζ cellular colocalization. (A-B) H1299 cells co-overexpressing the two proteins and imaged using an Olympus FV1000 microscope with a 60x objective. Scale bars: 10 µm. (C) A549 cells co-overexpressing the two proteins and imaged using a Zeiss Observer D1 with 63x objective. Scale bars: 10 µm.


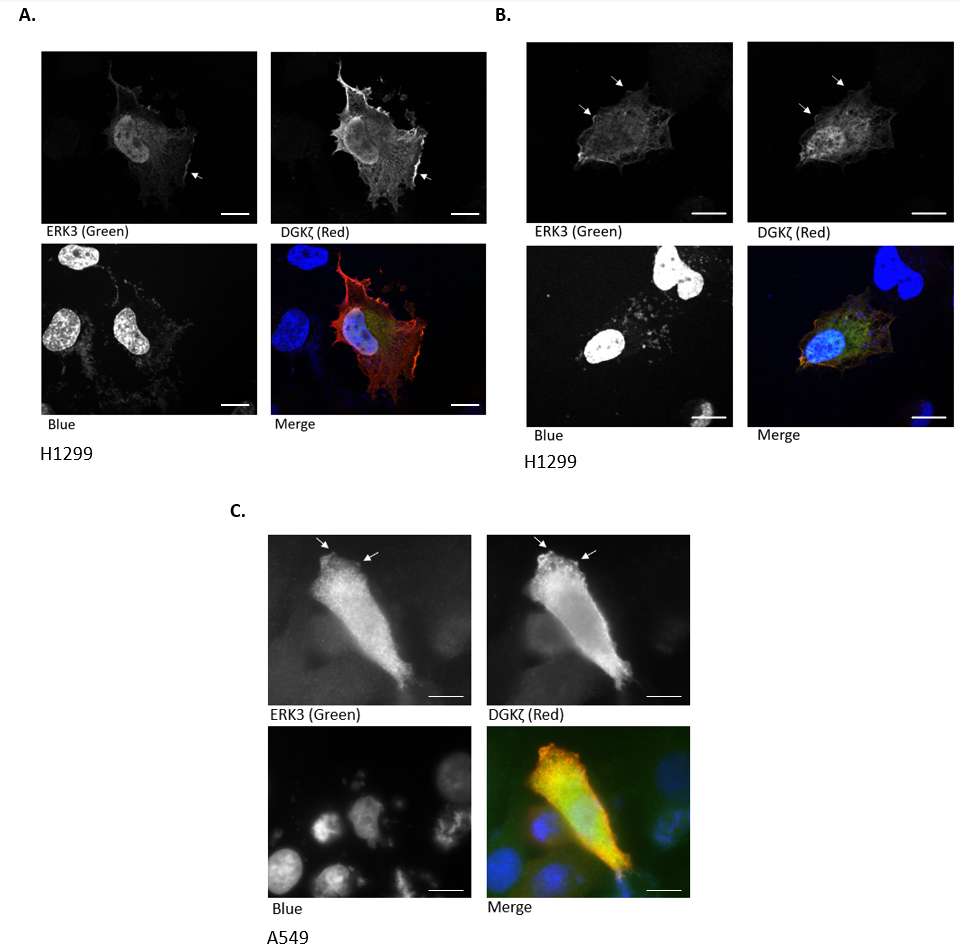


## Figure S3. ERK3 and DGKZ transcripts are negatively correlated in NSCLCs. Expression of MAPK6 (ERK3) (A) and DGKZ (B) mRNAs in lung adenocarcinomas (LUADs) or lung squamous carcinomas (LUSC) versus normal tissues. This analysis was performed on TCGA (tumor and normal samples) and GTEx datasets (normal samples) using GEPIA2 web server (Tang et al., 2017). (C-D) Correlation analysis of MAPK6 and DGKZ mRNA computed on cBioPortal (Cerami et al., 2012; Gao et al., 2013) using (C) LUAD patient samples (n=517, TCGA, Firehose Legacy dataset) or (D) LUSC patient samples (n=511, TCGA, Firehose legacy dataset).

**
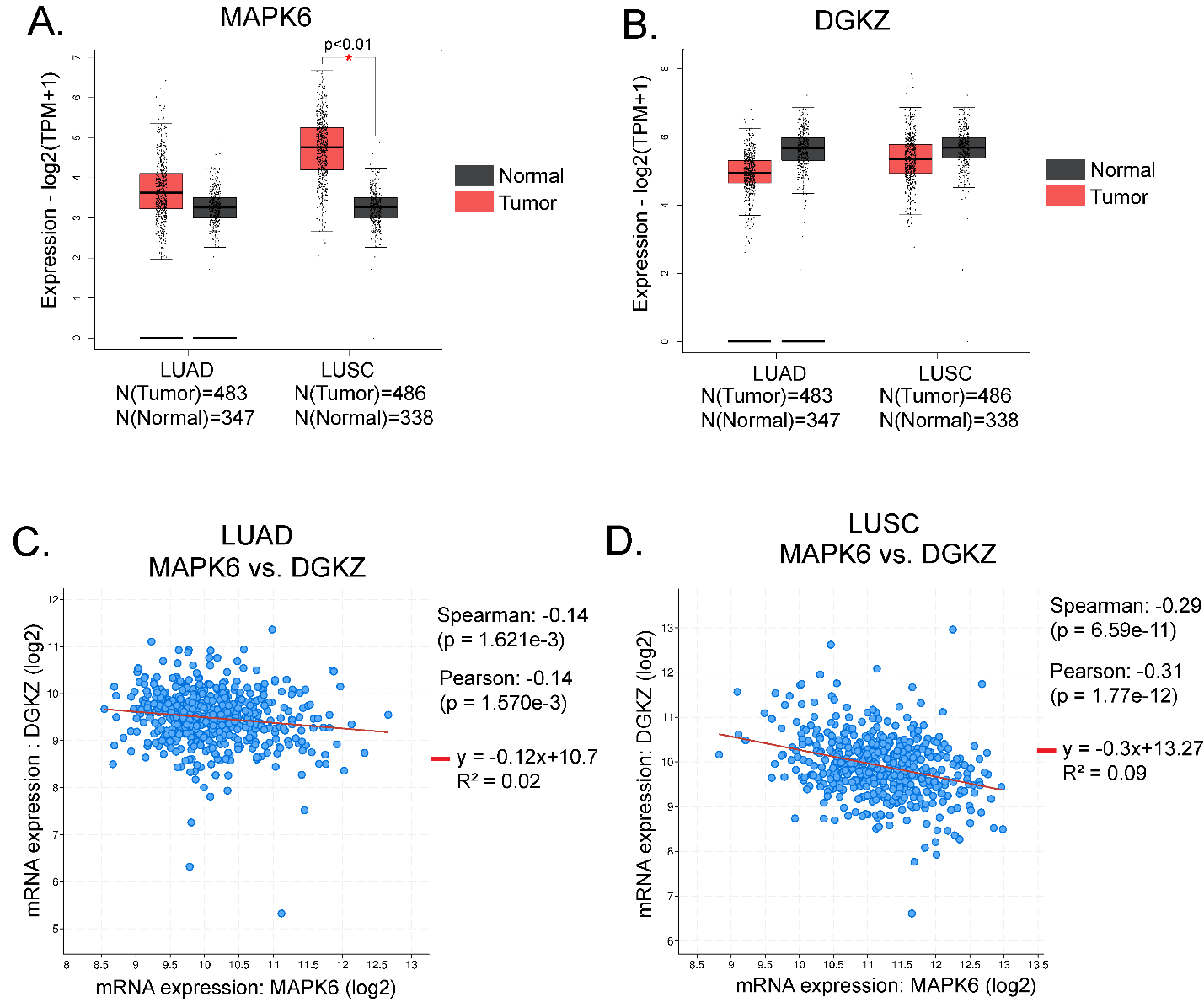
**

## Figure S4. DGKζ does not alter ERK3 phosphorylation. (A) Western blot analysis of ERK3 phosphorylation at Ser189 (P-S189) after ERK3 IP from A549 cells following transient transfection of a non-targeting, control siRNA (siCtrl) or a siRNA specifically targeting DGKζ. Expression levels of ERK3 and DGKζ in total cell lysates were also analyzed. (B) Flag-ERK3 was co-overexpressed with increasing amounts of HA-DGKζ in H1299 cells and ERK3 phosphorylation at Ser189 was assessed by Western blot. ImageJ was used to quantify ERK3 phosphorylation at Ser189 (P-S189) relative to total ERK3 level by densitometry.


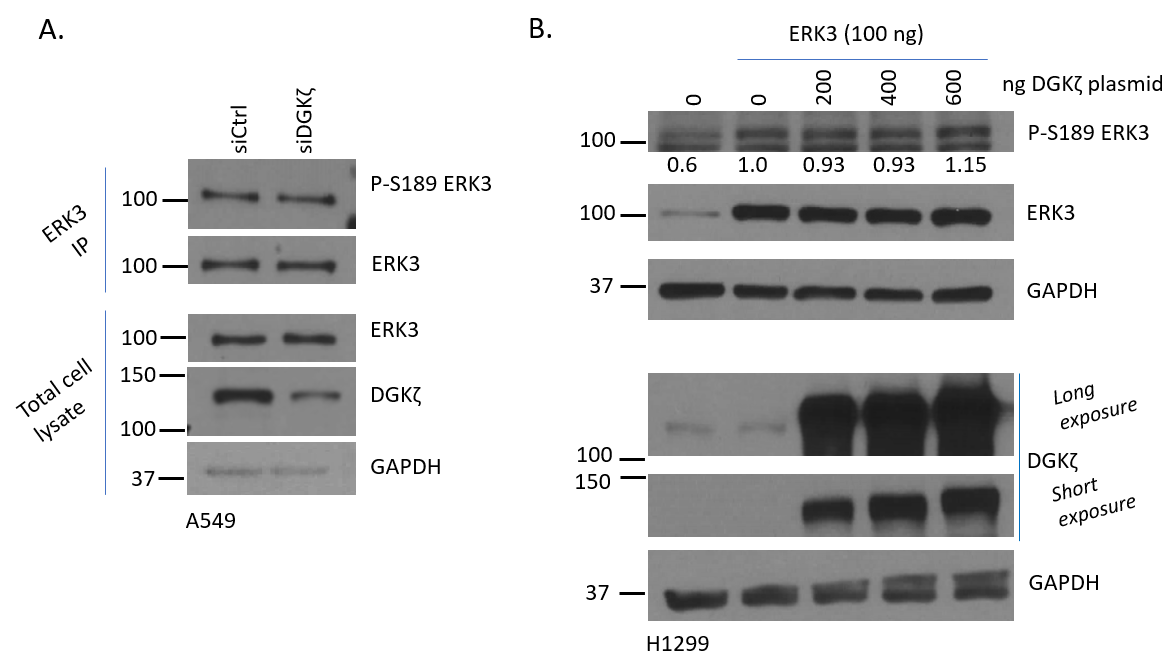


## Table S3. Chart of ERK3 accumulation scoring at cell membrane protrusions. Written description of scoring system for Figure 7 with examples of Flag epitope staining (for ERK3) representative of each category.

| **Table S3: Scoring** | | | |
| --- | --- | --- | --- |
| 1 | 2 | 3 | 4 |
|  |  |  |  |
| No discernible enrichment of ERK3 at outward curves of the membrane region as compared with the rest of the cell | ERK3 enrichment at one or more cell membrane protrusions which appears as a thin line and/or a very short protrusion containing moderate/high ERK3 enrichment | Moderately high ERK3 enrichment at one or more cell membrane protrusions which are usually at least 2x as thick (or ‘deep’) as the ’thin line’ observed with scoring 2 | High ERK3enrichment at one or more cell membrane protrusions which have higher intensity than cytosolic and nuclear ERK3 staining and have at least similar ‘thickness’ and length to scoring 3 |
| 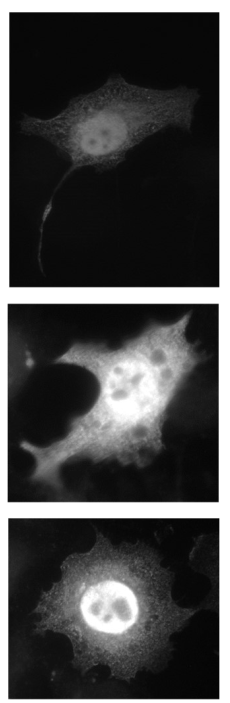 | 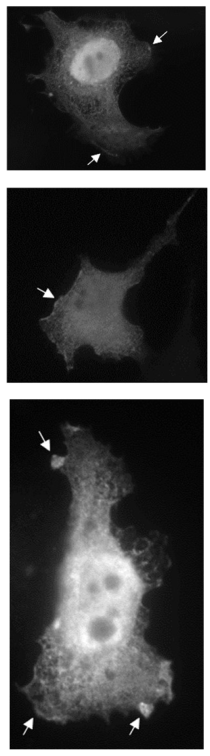 | 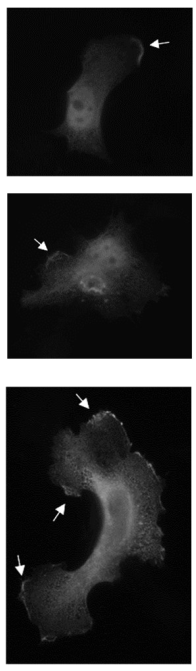 | 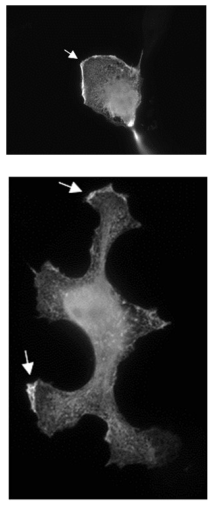 |
| Note: Images have been brightened 25% to promote visibility of the small images | | | |
